# Phase-Based Motor Skill Acquisition in Preschool Children with Different Participation Experience in a Kinesiology Program

**DOI:** 10.3390/jfmk11020133

**Published:** 2026-03-24

**Authors:** Kristian Plazibat, Tihomir Vidranski, Renata Barić

**Affiliations:** 1 Natural Sciences School Vladimir Prelog, 10000 Zagreb, Croatia; 2 Faculty of Educational Sciences, University of Slavonski Brod, 35000 Slavonski Brod, Croatia; 3 Faculty of Kinesiology, University of Zagreb, 10000 Zagreb, Croatiarenata.baric@kif.hr (R.B.)

**Keywords:** motor learning, phase-based learning, preschool children, motor competence, organized kinesiology program

## Abstract

**Background:** Early childhood is a critical period for the development of motor competence, which is closely related to later physical activity, educational readiness, and broader developmental outcomes. However, the temporal dynamics of motor skill acquisition in preschool children, particularly the time required to reach initial and early refinement phases of learning, remain insufficiently described. The aim of this study was to examine whether different levels of previous participation experience in an organized kinesiology program are associated with differences in the speed and quality of novel motor skill acquisition in preschool children, and to explore the relationship between baseline motor proficiency and phase-based indicators of motor learning. **Methods:** A total of 161 preschool children aged 5–6 years participated in the study and were grouped according to their previous participation experience in an organized kinesiology program (0 h, ~120 h, ~350 h, and ~470 h). Following BOT-2 assessment, all participants completed a standardized 7-week motor learning program that included nine previously unfamiliar motor tasks. Using a phase-based video analysis protocol, three learning indicators were recorded: time to Phase 1 (F1; first successful execution), time to Phase 2 (F2; initial refinement of performance), and final performance quality (K). Group differences and associations were first examined descriptively and correlationally, after which additional multivariable regression models were performed to determine whether previous participation experience and baseline motor proficiency were independently associated with motor learning outcomes. **Results:** The findings showed consistent differences across groups, with children who had greater previous participation experience generally reaching F1 and F2 more rapidly and achieving higher final performance quality scores. Higher BOT-2 scores were also associated with shorter learning times and better final performance quality. In the multivariable models, both previous participation experience in an organized kinesiology program and BOT-2 total score were independently associated with Phase 1 attainment time and final performance quality, whereas only previous participation experience remained independently associated with Phase 2 attainment time. The applied phase-based observational protocol demonstrated good to excellent inter-rater reliability across the evaluated motor learning variables. **Conclusions:** These findings provide phase-based temporal indicators of motor learning progression in preschool children and suggest that previous participation experience in an organized kinesiology program and baseline motor competence are meaningfully associated with the speed and quality of acquiring new motor tasks. The findings also demonstrate the potential of phase-based approaches for quantifying motor learning dynamics in early childhood settings. Such indicators may offer useful reference information for instructional pacing and the planning of motor learning activities, while also serving as practically relevant predictors for adapting future kinesiology programs to children’s motor readiness. Future research should further examine these relationships using longitudinal and analytically expanded designs.

## 1. Introduction

Motor development in early childhood is widely acknowledged as a fundamental marker of neuromuscular maturation and a crucial predictor of school readiness, long-term physical activity participation, and overall health trajectories [1,2]. In this sensitive developmental window, structured physical activity programs have been shown to enhance motor competence, coordination, muscular control, and postural balance [3,4,5]. These programs are especially important for developing motor readiness—a construct referring to a child’s preparedness to acquire, integrate, and refine novel motor skills [6].

However, despite well-documented developmental benefits, recent evidence suggests that a substantial proportion of children enter formal schooling with suboptimal levels of motor competence, often ranging from 9% to 52%, with less than half of preschoolers meeting recommended physical activity levels—a critical contributor to motor proficiency [7,8]. This phenomenon is particularly evident in underserved or low-resource communities, where opportunities for high-quality physical activity are limited [9]. As a result, early educational interventions often encounter a heterogeneous motor baseline among children, potentially undermining equal access to physical, cognitive, and academic development. Although numerous studies have confirmed the positive effect of structured physical activity on general motor proficiency [10,11], few have examined how prior experience in such programs affects the process of learning new motor tasks. Motor learning, as conceptualized in established frameworks, is a process that spans from initial acquisition, through cognitive structuring and associative refinement, ultimately progressing toward automatization [12]. Recent interventions suggest that well-structured development programs accelerate these learning trajectories, even in relatively short timeframes [13,14]. A growing body of evidence further suggests that early motor learning is not only trainable but also measurable with phase-sensitive methods [15]. Recent studies have introduced interventions that focus on early movement competencies and active play as tools for improving basic motor learning outcomes [16]. However, many of these programs remain limited in diagnostic resolution and fail to capture the pace children transition from one learning phase to the next. To address these limitations, the present study applies a phase-based video analysis protocol grounded in the motor learning model of Fitts and Posner [17], adapted by Neljak [18] for pedagogical and instructional use in school settings, and which the authors further adjusted for preschool populations while focusing on the first two phases. The method identifies two critical phases in real-time: gaining a structural understanding in Phase 1 (F1) and coordinated execution in Phase 2 (F2). By combining this framework with standardized motor competence assessment, the present study examines how previous participation in an organized kinesiology program and baseline motor competence relate to learning dynamics in preschool children. This study builds upon previous work [19] and aligns with contemporary pedagogical shifts toward individualized instruction and formative evaluation [20,21].

The aim of this study was to examine whether different levels of previous participation experience in an organized kinesiology program are associated with differences in the speed and quality of novel motor skill acquisition in preschool children, and to explore the relationship between baseline motor proficiency and phase-based indicators of motor learning. Task complexity was considered within the descriptive and interpretative framework of the study, as the applied motor tasks differed in their structural demands.

To address these aims more directly, the study combined descriptive, correlational, and multivariable analytical approaches in order to examine whether previous participation experience in an organized kinesiology program and baseline motor proficiency were independently associated with phase-based motor learning outcomes.

We expected that children with greater previous participation experience in an organized kinesiology program would generally progress more quickly through the observed learning phases and demonstrate higher final performance quality. We also expected that higher baseline motor proficiency would be associated with shorter learning times and higher final performance outcomes.

## 2. Materials and Methods

### 2.1. Participants

A total of 161 preschool children (aged 5–6 years; 83 girls, 78 boys) from kindergartens in the city of Zagreb participated in this study. Participants were classified into four groups according to their previous experience of participation in an organized kinesiology program: G1 (*n* = 40; no prior participation), G2 (*n* = 41; approximately 120 h), G3 (*n* = 41; approximately 350 h), and G4 (*n* = 39; approximately 470 h). Group allocation was therefore based on previous participation history and was non-randomized.

All children were healthy, attended regular preschool programs, and had no identified developmental delays. Sex distribution was comparable across the experience-based groups (χ^2^ = 5.205; df = 3; *p* = 0.157), indicating no statistically significant differences in gender representation between groups. Previous participation in the organized kinesiology program was determined based on institutional records of program attendance. Parental consent and institutional ethical approval were obtained prior to data collection.

### 2.2. Study Design

A non-randomized observational design was applied. All participants attended the regular preschool educational program, which represents the standard educational framework in the kindergarten setting. In addition to this regular program, some children had previous experience of participation in an organized kinesiology program, whereas others had no such prior participation. On this basis, participants were classified into four groups according to their previous experience of participation in the organized kinesiology program (G1: 0 h; G2: ~120 h; G3: ~350 h; G4: ~470 h). Group allocation was therefore based on previous participation history and was non-randomized.

Following baseline assessment, all participants completed the same new standardized 7-week motor learning protocol, which was applied equally across groups regardless of previous participation history. The purpose of this protocol was to observe phase-based motor learning progression under uniform instructional and environmental conditions. The program consisted of two sessions per week (45 min each), totaling 14 sessions, and included nine motor tasks that were unfamiliar to the children.

**Figure 1:**
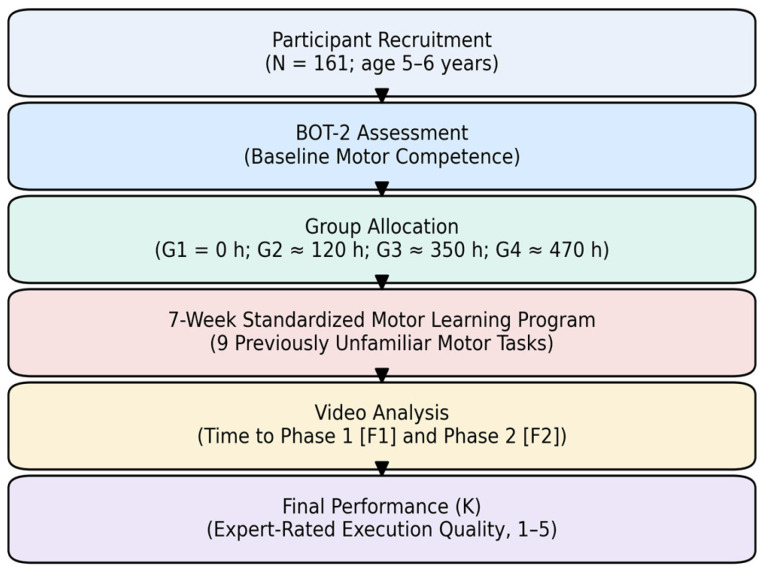
Flow diagram of the study design and procedure. The diagram presents the chronological sequence from participant recruitment and BOT-2 assessment to group allocation based on previous participation experience, the 7-week standardized motor learning program, video-based assessment of time to Phase 1 (F1) and Phase 2 (F2), and final performance (K) rated on a 5-point scale.

All sessions were delivered by licensed kinesiologists experienced in preschool settings. To support procedural consistency, instruction followed written guidelines and a brief practical orientation for instructors, and tasks were implemented using the same equipment, space organization, and progression rules across groups. Motor learning outcomes were derived from expert video analysis, quantifying the time to Phase 1 (F1), time to Phase 2 (F2), and final performance quality (K). Figure 1 presents the methodological sequence from participant inclusion to outcome assessment.

### 2.3. Instruments and Baseline Assessment

Motor proficiency was assessed using the Bruininks-Oseretsky Test of Motor Proficiency, Second Edition (BOT-2) Short Form [22]. This standardized test consists of 14 items drawn from 8 subtests and provides a composite score that reflects gross and fine motor performance. The BOT-2 test was administered individually by two trained evaluators. Inter-rater reliability exceeded ICC = 0.85.

### 2.4. Intervention Program

For the purposes of the present study, and immediately following BOT-2 assessment, all participants were exposed to a new standardized 7-week motor learning program applied under identical conditions across groups. This newly constructed program was independent of the regular kindergarten curriculum and also distinct from the previously attended kinesiology program. It was specifically designed to assess the speed and quality of acquiring new motor skills.

To ensure that the observed learning process reflected the acquisition of genuinely novel motor content, the program included motor tasks that were intentionally selected so that they had not been practiced within the regular preschool program or within the previously implemented kinesiology program. In this way, all participants, regardless of their previous experience of participation in structured exercise, performed the tasks as unfamiliar motor tasks. This allowed a standardized observation of how quickly and with what level of execution quality children acquired new motor tasks of different structural complexity.

The learning process was implemented through a total of 14 instructional sessions distributed over 7 weeks, according to a standardized procedure for all groups (see Supplementary Table S1). Motor learning outcomes were assessed through delayed expert video analysis based on predefined observational criteria (see Supplementary Table S2). The detailed phase-based assessment protocol and outcome definitions are presented in Section 2.5.

Within the newly constructed program, motor learning speed was additionally considered in relation to task complexity. A total of nine unfamiliar motor tasks of varying structural complexity were introduced. These included seven simple tasks, one complex task, and one more complex task. The simple motor tasks were: (1) jumping rope in place with a two-foot take-off, (2) jumping rope while moving forward, (3) tossing and catching a ball in place, (4) tossing and catching a ball while moving, (5) performing a two-foot vault over a gym bench from arm support, (6) carrying a 1 kg bag on the head, and (7) crawling through a hoop feet-first from a front-arm support. The complex task was the shoulder stand, and the more complex task was “Sambon Tsuki”, a karate three-strike forward movement pattern that required coordinated forward stepping combined with three consecutive arm strikes, while maintaining balance, spatial orientation, trunk stability, and sequential coordination of the upper and lower extremities. The complete program structure is presented in Supplementary Table S1.

Task performance was video recorded during the program according to a standardized recording procedure. At each session, two new motor tasks were practiced and recorded. The total effective execution time per task within a session was adjusted according to task complexity in order to allow an adequate observation window for phase attainment. Simple tasks were recorded for approximately 2 min per session, the complex task for approximately 3 min, and the more complex task for approximately 4 min. Across the full program, repeated task exposure was organized in accordance with the predefined program structure so that each motor task could be observed across multiple repetitions and instructional sessions.

Following completion of the final repetition of each motor task, final performance quality was assessed on the basis of the last recorded execution segment for that task. Final performance was scored on a 5-point scale by three independent expert raters using the same standardized observational criteria across all tasks and all participants.

### 2.5. Phase-Based Video Assessment Protocol

Motor learning was assessed through delayed expert video analysis using a phase-based observational protocol grounded in the motor learning model of Fitts and Posner [17] and adapted for pedagogical application by Neljak [18]. For the purposes of this study, only the first two learning phases were analyzed, as they represent the most developmentally appropriate and instructionally observable stages of motor skill acquisition in preschool children.

The following outcomes were defined: Phase 1 (F1), defined as the time (in seconds) until the first successful execution of the movement according to predefined task-specific criteria, indicating that the child had established a recognizable basic movement structure; Phase 2 (F2), defined as the time (in seconds) until the child demonstrated a more stable, coordinated, and repeatable execution pattern, reflecting initial refinement of the movement structure according to predefined task-specific criteria; and Final Performance (K), defined as expert-rated execution quality on a 5-point Likert scale.

The operational criteria used to determine phase attainment were defined in advance for each motor task and are provided in Supplementary Table S2. These criteria allowed expert raters to determine the exact point at which a child reached the initial acquisition phase (F1) and the initial refinement phase (F2) during the recorded execution sequence. Phase attainment was recorded only when the visible execution met the predefined observational criterion for the respective task.

Final performance quality (K) was assessed after completion of the final repetition of each motor task. The 5-point rating scale was based on the overall recognizability of the movement pattern, the coordination of the essential movement segments, movement control and stability, fluency of execution, and the overall quality of task performance. Higher scores indicated a more coordinated, stable, and technically recognizable execution.

Video recordings were reviewed by three independent kinesiologists, each with more than 5 years of professional experience in conducting physical activity programs for preschool children. Each task was analyzed separately for each participant with regard to phase transition timing and final performance quality. Expert consensus was used to confirm phase attainment, and disagreements exceeding 1 point in final performance scoring were resolved through subsequent discussion.

Inter-rater reliability of the phase-based performance assessment was evaluated using intraclass correlation coefficients (ICC) calculated for phase transition times and final performance ratings. The obtained coefficients indicated good to excellent agreement between evaluators, supporting the consistency of the applied observational procedure.

The theoretical framework applied in this study is based on the classic three-stage model proposed by Fitts and Posner, which distinguishes between the cognitive, associative, and autonomous stages of motor skill acquisition [17]. Neljak later adapted this framework for instructional use in physical education, reformulating it into a five-phase pedagogical model [18]. In the present study, only the first two adapted phases were used, as they correspond to the establishment of the basic movement structure and its initial functional refinement, which were the primary focus of the present phase-based analysis in preschool children.

Accordingly, in the present study, the cognitive stage was operationalized as Phase 1 (F1), reflecting the establishment of a recognizable basic movement structure, while the associative stage was operationalized as Phase 2 (F2), reflecting the initial coordination and refinement of execution.

### 2.6. Statistical Analysis

Descriptive statistics were calculated for all outcome variables and are presented as means, standard deviations, medians, interquartile ranges, and minimum–maximum values, depending on the nature of the variable. Normality of distribution was tested using the Kolmogorov–Smirnov test prior to inferential analysis. As several variables showed deviations from normal distribution, non-parametric statistical procedures were applied in the subsequent analyses.

Group differences in phase-based motor learning outcomes, including time to Phase 1 (F1), time to Phase 2 (F2), and final performance quality (K), were analyzed using the Kruskal–Wallis test, followed by the Conover–Iman post hoc test when appropriate. Spearman’s rank correlation coefficient was used to assess relationships between BOT-2 scores and motor learning variables.

In the descriptive interpretation of selected results, BOT-2 total scores were also considered in relation to normative categories provided in the BOT-2 manual, in order to contextualize differences in motor competence within the studied sample. Statistical significance was set at *p* < 0.05. All analyses were performed using IBM SPSS Statistics v.27.

#### Multivariable Analysis

To further align the analytical framework with the study aims, additional multivariable analyses were conducted to examine whether previous participation experience and baseline motor competence were independently associated with phase-based motor learning outcomes. For each participant, aggregated mean values across the nine motor tasks were calculated for time to Phase 1 (F1), time to Phase 2 (F2), and final performance quality (K). Separate regression models were then estimated for each outcome. Previous participation experience was entered as cumulative exposure to the organized kinesiology program (0, 120, 350, and 470 h), while BOT-2 total score, age, and sex were simultaneously included as predictors.

Because the distributions of F1 and F2 attainment times were positively skewed, log-transformed outcomes [log(time + 1)] were used for these models. Final performance quality (K) was analyzed using linear regression. Regression coefficients, standard errors, *p*-values, and adjusted R^2^ values were reported. These additional analyses were intended to determine whether previous participation experience and baseline motor competence contributed independently to phase-based learning outcomes beyond the descriptive group comparisons and bivariate associations.

## 3. Results

### 3.1. Group Differences in Phase-Based Motor Learning Outcomes

Descriptive statistics indicated clear group-level differences in time to Phase 1 (F1), time to Phase 2 (F2), and aggregated final performance quality (K) across the four groups (Table 1). Children in G3 and G4, who had greater previous experience of participation in the organized kinesiology program, reached the first two learning phases faster and achieved higher final performance scores than children in G1 and G2.

Table 1 summarizes the mean values for F1, F2, and the aggregated final performance quality score (K) across the four groups. Overall, the pattern of results showed more favorable phase-based learning outcomes in children with greater previous participation experience, particularly in relation to the speed of phase attainment and the overall quality of final task execution. Statistical analysis confirmed that the differences among groups were significant for all three outcomes (*p* < 0.001). To further examine between-group differences, Conover–Iman post hoc comparisons with adjusted *p*-values were performed, and the results are presented in Table 2.

### 3.2. Pairwise Comparisons Between Groups

As shown in Table 2, post hoc comparisons confirmed that children in G3 and G4 generally demonstrated more favorable phase-based learning outcomes than children in G1 and G2. The most consistent differences were observed between G1 and the more experienced groups (G3 and G4), as well as between G2 and G4.

Differences between G3 and G4 were smaller in magnitude. In this comparison, no statistically significant differences were observed for F1 and F2, while a significant difference remained present for final performance quality (K), indicating that the two most experienced groups showed relatively similar learning speed but differed to some extent in final execution quality.

Overall, the pairwise comparisons support the interpretation that greater previous experience of participation in the organized kinesiology program was associated with more efficient progression through the observed learning phases and with higher final performance quality.

### 3.3. Phase-Based Learning Times by Task Complexity and BOT-2 Category

To further contextualize the observed differences in motor learning, Table 3 presents descriptive indicators of the time required to reach Phase 1 (F1) and Phase 2 (F2) across three task complexity levels (simple, complex, and more complex), stratified according to BOT-2 motor proficiency category (Average vs. Above Average).

As shown in Table 3, children in the Above Average BOT-2 category reached both learning phases faster than children in the Average category across all levels of task complexity. This pattern was most pronounced in simple tasks and remained visible, although less distinct, as task complexity increased. In addition to shorter learning times, the Above Average group generally showed narrower interquartile ranges, indicating more consistent performance within that category.

These descriptive results suggest that higher baseline motor competence was associated with faster phase attainment across unfamiliar motor tasks, while increasing task complexity was associated with longer learning times in both proficiency categories.

### 3.4. Descriptive Visualization of Learning Time by Task Complexity and BOT-2 Category

**Figure 2:**
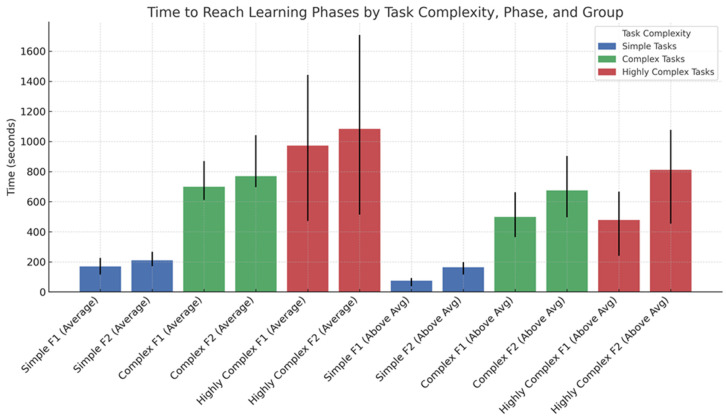
Descriptive visualization of the time required to reach Phase 1 (F1) and Phase 2 (F2) in novel motor skill acquisition by task complexity and BOT-2 motor proficiency category. Bars represent mean learning times in seconds. Error bars indicate the interquartile range (25th–75th percentile) for each condition. Task complexity is grouped as Simple, Complex, and More Complex. Phase 1 refers to the initial structural acquisition of the task, whereas Phase 2 refers to refined, repeatable execution.

Figure 2 illustrates the descriptive pattern of mean learning times for Phase 1 (F1) and Phase 2 (F2) across task complexity levels and BOT-2 motor proficiency categories. The figure visually supports the general tendency observed in Table 3: learning times increased with task complexity, while children classified in the Above Average BOT-2 category reached both phases more quickly than children in the Average category. The visual difference between proficiency groups was more apparent in simple tasks, whereas in more complex tasks the gap between groups appeared smaller. This descriptive pattern suggests that baseline motor competence may be more strongly reflected in the early acquisition of less structurally demanding tasks, while increasing task demands may reduce the visible separation between groups.

### 3.5. Associations Between Baseline Motor Competence and Phase-Based Learning Indicators

Spearman’s rank-order correlations were used to examine associations between baseline motor competence (BOT-2 composite score) and phase-based motor learning indicators. Higher BOT-2 scores were strongly associated with shorter time to reach Phase 1 (F1) and Phase 2 (F2), indicating faster acquisition and early refinement of novel motor tasks. In addition, BOT-2 scores were positively associated with higher aggregated final performance quality (K). The correlation coefficients and significance levels are presented in Table 4.

As shown in Table 4, baseline motor competence (BOT-2 composite score) was strongly and inversely associated with the time required to reach Phase 1 (F1; ρ = −0.73, *p* < 0.001) and Phase 2 (F2; ρ = −0.61, *p* < 0.001), indicating that children with higher motor proficiency progressed more rapidly through the early stages of motor learning. In contrast, BOT-2 scores were positively associated with aggregated final performance quality (K; ρ = 0.59, *p* < 0.001), suggesting that greater baseline competence was related not only to faster phase attainment but also to higher-quality execution of the newly learned motor tasks.

To further examine whether previous participation experience in an organized kinesiology program and baseline motor proficiency were independently associated with motor learning outcomes, additional multivariable analyses were conducted.

### 3.6. Multivariable Associations with Learning Outcomes

To determine whether previous participation experience in an organized kinesiology program and baseline motor proficiency were independently associated with motor learning outcomes, additional multivariable regression models were performed. For each participant, aggregated mean values across the nine motor tasks were calculated for F1 attainment time, F2 attainment time, and final performance quality.

For Phase 1 attainment time, both previous participation experience in an organized kinesiology program and BOT-2 total score were significant independent predictors. Greater previous participation experience in an organized kinesiology program was associated with shorter time to reach F1 (B = −0.0014, *p* < 0.001), while higher BOT-2 scores were also associated with faster F1 attainment (B = −0.0162, *p* < 0.001). Age and sex were not significant predictors. The model explained 46.3% of the variance in Phase 1 attainment time (adjusted R^2^ = 0.463).

For Phase 2 attainment time, previous participation experience in an organized kinesiology program remained a significant independent predictor (B = −0.0010, *p* = 0.005), whereas BOT-2 total score was no longer significantly associated with F2 attainment time after adjustment for the other predictors (B = 0.0088, *p* = 0.303). Age and sex were not significant. The explanatory power of this model was modest (adjusted R^2^ = 0.035).

For final performance quality, both previous participation experience in an organized kinesiology program and BOT-2 total score were significant independent predictors. Greater previous participation experience in an organized kinesiology program was associated with higher final performance quality (B = 0.0034, *p* < 0.001), and higher BOT-2 scores were likewise associated with better final performance quality (B = 0.0684, *p* < 0.001). Age and sex were not significant predictors. This model explained 58.2% of the variance in final performance quality (adjusted R^2^ = 0.582).

Taken together, these multivariable findings indicate that previous participation experience in an organized kinesiology program and baseline motor proficiency contributed differently across learning outcomes. Both were independently associated with initial acquisition and final performance quality, whereas only previous participation experience in an organized kinesiology program remained independently associated with time to early refinement.

As shown in Table 5, the multivariable regression models revealed that previous participation experience in the organized kinesiology program and baseline motor proficiency were differentially associated with the observed learning outcomes. For Phase 1 attainment time, both previous participation experience in the organized kinesiology program and BOT-2 total score emerged as significant independent predictors, indicating that children with greater prior participation experience and higher baseline motor competence reached the initial acquisition phase more quickly (*p* < 0.001). For Phase 2 attainment time, however, only previous participation experience in the organized kinesiology program remained a significant predictor after adjustment (*p* = 0.005), whereas BOT-2 total score was no longer independently associated with the outcome (*p* = 0.303).

These findings suggest that baseline motor competence was more strongly related to the speed of initial phase attainment, whereas previous participation experience in the organized kinesiology program was more consistently associated with progression through later early learning stages. For final execution quality, both previous participation experience in the organized kinesiology program and BOT-2 total score remained significant independent predictors, indicating that prior participation experience and baseline motor proficiency jointly contributed to better motor performance (*p* < 0.001).

## 4. Discussion

The present study showed that greater previous experience of participation in organized kinesiology program was associated with more favorable phase-based motor learning outcomes in preschool children. Specifically, children with 350 h or more of previous program experience (G3 and G4) progressed more rapidly through Phase 1 (F1) and Phase 2 (F2) and achieved higher final performance quality than children with less or no previous participation experience. These findings are consistent with previous research suggesting that fundamental movement skills form an important foundation for later physical development, movement competence, and sustained physical activity participation [23,24].

A key contribution of this study lies in the phase-based quantification of the time required to reach observable stages of motor skill acquisition in unfamiliar tasks. When performance was described according to BOT-2 normative categories [23], children in the Average category required up to 226 s to reach Phase 1 and 267 s to reach Phase 2 in simple tasks, whereas these values increased markedly as task complexity increased. For more complex motor tasks, children in the Average category required substantially more time, with the longest intervals observed in tasks of greater structural complexity. In contrast, children in the Above Average category reached both phases in shorter time intervals across all levels of task complexity. Importantly, these findings should not be interpreted as universal normative standards, but rather as phase-based temporal indicators obtained within the present research context that may serve as useful reference information when planning motor learning activities, instructional pacing, and progression in early childhood settings.

For practical interpretation, these descriptive ranges represent effective practice time (time-on-task). The values in Table 3 correspond to approximately 1.9–3.8 min to reach Phase 1 in simple tasks for the Average BOT-2 category (116–226 s) versus 0.6–1.5 min in the Above Average category (39–92 s). For Phase 2, the corresponding ranges are approximately 2.9–4.5 min (171–267 s) vs. approximately 1.9–3.3 min (115–199 s), respectively.

Percentile-based descriptive indicators further clarified individual variability in learning progression. For example, 75% of children in the Average BOT-2 category reached Phase 1 in simple tasks within 226 s, whereas children in the Above Average category generally required less time. From a practical standpoint, such distribution-based information may help educators and kinesiologists estimate approximate learning intervals, adapt repetition dosage, and better align task progression with the child’s current level of readiness.

An important descriptive finding was that children classified in the Above Average BOT-2 category were primarily represented by participants with previous participation experience in the organized kinesiology program, whereas children without such experience were primarily represented in the Average category. Although this pattern should not be interpreted causally, it supports the broader assumption that previous structured movement experience may be linked to more developed baseline motor competence and, consequently, to more efficient early learning of unfamiliar motor tasks.

Importantly, the correlational analysis (Table 4) further supports the relationship between baseline motor competence and phase-based learning dynamics. Higher BOT-2 composite scores were strongly associated with shorter time to Phase 1 (ρ = −0.73, *p* < 0.001) and Phase 2 (ρ = −0.61, *p* < 0.001), and were moderately positively associated with higher aggregated final performance quality (K) (ρ = 0.59, *p* < 0.001). These findings indicate that children with higher baseline motor competence tended to establish the basic movement structure and reach early refinement more efficiently, while also achieving higher execution quality across tasks. Given the observational exposure-group design, these correlations should be interpreted as associations and may partly reflect shared variance and confounding with previous participation experience.

A further contribution of the revised analysis is that it enables simultaneous evaluation of previous participation experience in an organized kinesiology program and baseline motor proficiency. This distinction is methodologically important because descriptive group comparisons and bivariate correlations alone cannot determine whether differences in motor learning outcomes are independently associated with previous participation experience or instead reflect pre-existing differences in motor competence. In the present multivariable models, both previous participation experience in an organized kinesiology program and BOT-2 total score remained independently associated with Phase 1 attainment time and final performance quality, suggesting that previous participation experience and baseline motor proficiency make distinct contributions to early motor learning. By contrast, only previous participation experience in an organized kinesiology program remained independently associated with Phase 2 attainment time, which may indicate that progression into early refinement is more strongly related to accumulated structured practice than to baseline general motor proficiency alone.

This finding adds nuance to the interpretation of the learning process in early childhood. Initial acquisition appears to depend on both the child’s pre-existing motor resources and previous participation experience in an organized kinesiology program, whereas early refinement may be shaped more strongly by accumulated exposure to organized movement practice. In practical terms, this suggests that baseline motor proficiency may be especially relevant for how quickly children grasp the basic structure of unfamiliar tasks, while sustained previous participation experience in an organized kinesiology program may play a more important role in stabilizing and refining performance over repeated practice.

From an applied perspective, these findings may be particularly relevant during the transition from preschool to formal schooling. Identifying systematic differences in motor competence and phase-based learning progression may provide useful support for early childhood educators and physical education specialists. First, the results may contribute to more developmentally appropriate preparation of children for structured physical education content in early school years. Second, they may support refinement of motor learning plans based on observed learning dynamics, thereby contributing to more individualized and goal-oriented instruction. In this sense, linking motor readiness with observable learning progression offers practically relevant information for planning physical literacy pathways during a sensitive developmental period.

The present study also contributes to the literature by shifting the focus from endpoint evaluation to process-sensitive tracking of motor learning. Previous studies, including the CHAMPS-DK trial, have shown that structured physical activity is associated with improved coordination and physical fitness in children [25]. In particular, Bugge et al. [26] demonstrated that three years of enhanced physical education improved cardiorespiratory fitness and motor coordination, supporting the developmental relevance of structured activity exposure. However, although such studies provide important evidence on motor and fitness outcomes, they do not usually address the temporal progression through specific learning phases of new motor tasks. By applying the theoretical framework of Fitts and Posner [17], together with its pedagogical adaptation by Neljak [18], the present study extends this line of work by examining how quickly children progress through early observable stages of learning.

Recent studies also support the view that motor learning in early childhood is highly adaptable and responsive to structured interventions. Kambas and Venetsanou [27] showed that even an innovative online fairytale-based intervention could improve motor proficiency in kindergarten children, emphasizing the adaptability of neuromotor systems in young children. Similarly, Wedderkopp et al. [28] reported significant improvements in motor skills after a 12-week goal-oriented play intervention, again supporting the value of targeted and developmentally appropriate movement experiences. These findings are in line with the present results and reinforce the premise that early motor learning is responsive to structured and purposeful practice.

Our findings further align with studies emphasizing the plasticity of motor learning in preschool children. Ruiz-Esteban et al. [13] reported measurable improvements in gross motor development following a focused intervention, while Spring et al. [29] showed that eight weeks of active play improved basic motor competencies. Although these studies demonstrated beneficial effects on motor outcomes, they did not examine the speed of progression through early learning phases. In that sense, the present study adds a process-oriented dimension by describing not only performance outcomes, but also how learning unfolds over time.

A further conceptual implication of the present findings may be considered in light of the proficiency barrier framework. As discussed by Brian et al. [30], overcoming early barriers in motor competence is important because it may facilitate broader developmental progression and support later participation, confidence, and more complex movement engagement. The present findings support this perspective by showing that children with more favorable baseline motor competence and greater previous movement experience generally progressed more efficiently through early learning phases. In practical terms, this suggests that strengthening early motor competence may support children’s readiness for subsequent and more demanding motor learning experiences.

Relatedly, the educational significance of early motor proficiency may extend beyond immediate performance outcomes. Trecroci et al. [31] highlighted that enriched movement-related experiences may also be associated with higher-order developmental outcomes such as motor creativity. Although motor creativity was not examined in the present study, the current findings may be viewed as relevant within that broader developmental perspective, as more efficient early acquisition of movement structure and refinement may help create a stronger basis for later exploratory and adaptive motor behavior.

From a pedagogical standpoint, the descriptive data carry relevant practical implications. For example, children with Average baseline motor proficiency generally required more time to reach early coordination and refinement in unfamiliar tasks, whereas children with Above Average baseline proficiency progressed more rapidly. As task complexity increased, the difference between groups appeared to narrow, which may indicate that higher task demands reduce the visible differentiation between children or impose stronger structural constraints on performance.

This pattern suggests that simpler tasks may allow greater instructional individualization based on baseline motor competence, whereas more complex tasks may require more universally scaffolded practice conditions. In this regard, previous studies also support the value of context-sensitive pedagogy, showing that preschool children respond differently to movement interventions depending on age, task structure, and cognitive-developmental characteristics [32,33]. Taken together, these findings reinforce the importance of adaptive instruction, formative observation, and progressive task design in early motor learning.

The present findings also support a more dynamic and feedback-responsive instructional approach. By describing approximate phase-based learning intervals, the study may help practitioners judge when additional repetition is needed, when a child may be ready for corrective feedback, and when progression toward a more demanding task variant may be appropriate. This interpretation is compatible with the OPTIMAL theory of motor learning [34], which emphasizes the role of motivation, attentional focus, and appropriately structured practice in accelerating motor learning.

Despite these contributions, several limitations should be acknowledged. First, the study was based on an observational exposure-group design, and participants were not randomly assigned to groups; therefore, the findings should be interpreted in terms of associations rather than causal effects. Second, the study focused only on the first two learning phases (F1 and F2), while later stages such as stabilization, retention, and automatization were not examined. In addition, Phase 2 represents a sequentially dependent stage that can only occur after Phase 1 has been attained. From an analytical perspective, a multi-state time-to-event framework would provide the most advanced representation of this dependency. However, the present multivariable regression approach was adopted as a practical and feasible compromise to better align the analysis with the stated study aims. Future studies should therefore consider sequential or multi-state modeling strategies to examine phase dependency and motor learning progression in greater detail. Third, although the expert video-analysis procedure demonstrated good to excellent inter-rater reliability, the observational approach still depends on expert judgment, and future studies may benefit from integrating sensor-based or instrument-supported assessments to strengthen objectivity. Fourth, the sample was naturally mixed by sex and drawn from one local context, which should be considered when interpreting generalizability. Finally, although task complexity was included descriptively, future studies using longitudinal and more advanced analytical designs could further clarify how baseline competence, prior experience, and task demands interact over time.

Future research should therefore build on these findings by examining later phases of motor learning, retention effects, and longer-term developmental transfer. Longitudinal designs would be particularly valuable in determining whether the observed differences in early learning speed are associated with more stable motor competence over time. In addition, future studies should examine whether early advantages in phase-based learning are related to later outcomes such as motor creativity, task adaptability, and broader engagement in physical activity.

In summary, the present study provides a process-oriented contribution to the understanding of motor learning in preschool children. By integrating previous participation experience in an organized kinesiology program, baseline motor competence, and phase-based observation of unfamiliar motor tasks, the study extends existing outcome-focused approaches and offers practically relevant information for pedagogical planning in early physical education and kinesiology programs. Importantly, the additional multivariable findings suggest that previous participation experience in an organized kinesiology program and baseline motor proficiency may also function as meaningful predictors of early learning dynamics. In practical terms, these indicators may help educators and kinesiologists better anticipate learning pace, differentiate task progression, and adapt instructional conditions to the child’s current level of motor readiness.

## 5. Conclusions

This study examined phase-based motor learning outcomes in preschool children in relation to previous participation experience in an organized kinesiology program and baseline motor competence assessed by the BOT-2 test. The findings indicate that children with greater previous participation experience generally progressed more efficiently through the early observable phases of learning unfamiliar motor tasks and achieved higher final performance quality.

Children with more previous participation experience in an organized kinesiology program reached Phase 1 (initial structural acquisition) and Phase 2 (initial refinement) in shorter time intervals than children with less or no previous participation experience. Similarly, children with higher baseline motor competence demonstrated more favorable phase-based learning progression across tasks of different structural complexity.

A specific contribution of the study lies in the application of a phase-based observational framework that extends beyond end-point performance and provides process-oriented insight into how preschool children progress through early learning stages, as well as the level of final performance quality they achieve, in relation to their differing previous participation experience in an organized kinesiology program.

The additional multivariable analyses further strengthened the interpretation of these findings by showing that previous participation experience in an organized kinesiology program and baseline motor competence were differentially associated with the observed learning outcomes. Both predictors were independently associated with Phase 1 attainment and final performance quality, whereas only previous participation experience remained independently associated with Phase 2 attainment time. These findings suggest that initial acquisition may depend on both baseline motor resources and prior structured experience, while early refinement may be more consistently related to accumulated organized practice.

The observed time intervals should not be interpreted as universal normative benchmarks, but rather as context-specific phase-based temporal indicators derived from the present sample. As such, they may provide useful reference information for planning instructional pacing, repetition dosage, and task progression in preschool physical education and kindergarten kinesiology practice.

From an applied perspective, the findings support the relevance of considering both participation experience in an organized kinesiology program and baseline motor competence when designing early motor learning activities. In particular, these variables may serve as practically relevant predictors when planning future kinesiology programs for preschool children, helping educators and kinesiologists better anticipate learning pace, individualize task progression, and adapt instructional conditions to the child’s current level of motor readiness. However, because the study was based on an observational, non-randomized design, the findings should be interpreted as associations rather than causal effects.

Overall, the present study supports the value of a phase-based approach to early motor learning and contributes additional insight into how previous participation experience in an organized kinesiology program and baseline motor competence are associated with the speed and quality of acquiring unfamiliar motor tasks in preschool children.

## Figures and Tables

**Table 1:** Time to reach Phase 1 (F1) and Phase 2 (F2), and final performance quality score (K), across four participant groups (G1–G4) defined by previous experience of participation in the organized kinesiology program. Mean and standard deviation (SD) are presented for F1 and F2. K represents the aggregated mean final performance quality score across the nine analyzed motor tasks. F1 represents the initial successful execution of the task (motor structuring), F2 denotes repeatable refined performance (early refinement), and K is the expert-rated final performance quality score on a 5-point Likert scale.

Group	F1 Mean ± SD (s)	F2 Mean ± SD (s)	K Mean (1–5)
G1	137.6 ± 75.0	120.8 ± 99.0	2.04
G2	138.5 ± 56.3	204.8 ± 37.5	3.20
G3	51.6 ± 34.8	163.1 ± 57.9	3.83
G4	53.0 ± 29.7	113.6 ± 44.7	4.42

**Table 2:** Pairwise comparisons (Bonferroni-adjusted *p*-values) for time to Phase 1 (F1), time to Phase 2 (F2), and final execution score (K) across groups (G1–G4). All comparisons are based on Conover-Iman post hoc tests following Kruskal–Wallis analysis. Statistically significant differences (*p* < 0.05) indicate pairwise group differences in phase-based learning outcomes.

Comparison	F1 Time	F2 Time	Final Score
G1 vs. G2	0.041	0.022	0.034
G1 vs. G3	<0.001	<0.001	<0.001
G1 vs. G4	<0.001	<0.001	<0.001
G2 vs. G3	0.004	0.001	0.016
G2 vs. G4	<0.001	<0.001	<0.001
G3 vs. G4	0.128	0.067	0.042

**Table 3:** Time required (in seconds) to reach Phase 1 (F1) and Phase 2 (F2) in novel motor skill acquisition, stratified by task complexity (Simple, Complex, More Complex) and BOT-2 motor proficiency category (Average vs. Above Average). Displayed are mean values, standard deviations (SD), medians, minimum and maximum values, and interquartile ranges (25th and 75th percentiles). F1 denotes initial structural acquisition of the motor task; F2 denotes refined, repeatable execution.

Task Complexity	Phase	BOT-2 Category	Mean	SD	Median	Min	Max	P25–P75
Simple	F1	Average	169.9	64.5	192.2	56.8	259.8	116.1–226.5
Simple	F2	Average	210.2	70.5	226.5	0.0	294.4	171.3–267.2
Complex	F1	Average	699.1	248.8	727.0	0.0	1014.3	611.2–870.3
Complex	F2	Average	769.4	389.0	960.8	0.0	1076.0	696.0–1042.3
More Complex	F1	Average	973.2	564.2	1024.3	0.0	1863.3	471.2–1443.5
More Complex	F2	Average	1083.7	677.4	1178.8	0.0	1882.7	513.2–1708.2
Simple	F1	Above Average	75.2	54.3	57.3	13.4	225.5	38.6–92.2
Simple	F2	Above Average	164.4	62.7	166.1	44.2	292.8	115.4–198.7
Complex	F1	Above Average	498.4	197.9	503.7	76.3	904.0	365.0–662.3
Complex	F2	Above Average	674.9	273.7	726.0	11.3	1076.7	496.0–903.7
More Complex	F1	Above Average	478.8	416.5	393.0	0.0	1683.7	240.0–667.3
More Complex	F2	Above Average	812.0	488.4	757.3	0.0	1861.0	454.3–1077.3

**Note:** In the present sample, the Average BOT-2 category largely comprised children without previous participation in the organized kinesiology program, whereas the Above Average category predominantly comprised children with previous participation experience. Values of 0.0 indicate that Phase 2 was not attained within the observation window and were coded as 0 for descriptive purposes.

**Table 4:** Spearman’s correlations between baseline motor competence (BOT-2 composite score) and phase-based motor learning indicators (N = 161).

Variable Pair	ρ (Spearman)	*p*-Value
BOT-2 vs. Phase 1 time (F1)	−0.73	<0.001
BOT-2 vs. Phase 2 time (F2)	−0.61	<0.001
BOT-2 vs. Final performance quality (K)	0.59	<0.001

**Note:** BOT-2 denotes the composite score from the BOT-2 Short Form. F1 and F2 denote time (s) to Phase 1 and Phase 2, respectively. K denotes the aggregated final performance quality score across the nine motor tasks.

**Table 5:** Multivariable regression models for phase-based motor learning outcomes.

Outcome Variable	Predictor	B	SE	*p*
log(F1 + 1)	Exposure hours	−0.0014	0.0002	<0.001
	BOT-2 total score	−0.0162	0.0041	<0.001
	Age	0.0017	0.0043	0.698
	Sex (male)	−0.0661	0.058	0.256
	**Adjusted R^2^**	**0.463**		
log(F2 + 1)	Exposure hours	−0.001	0.0004	0.005
	BOT-2 total score	0.0088	0.0085	0.303
	Age	0.0086	0.0089	0.338
	Sex (male)	−0.0365	0.1203	0.762
	**Adjusted R^2^**	**0.035**		
Final execution quality	Exposure hours	0.0034	0.0004	<0.001
	BOT-2 total score	0.0684	0.0101	<0.001
	Age	0.0068	0.0107	0.526
	Sex (male)	0.2303	0.1425	0.108
	**Adjusted R^2^**	**0.582**		

**Note:** Exposure hours were coded as cumulative previous participation in structured physical activity (0, 120, 350, and 470 h). Phase timing outcomes were modeled using log-transformed values [log(time + 1)] due to positive skewness. Sex was coded as a binary variable, with male entered as the comparison category. **B** = unstandardized regression coefficient; **SE** = standard error; ***p*** = significance level; **Adjusted R^2^** = proportion of explained variance adjusted for the number of predictors.

## Data Availability

The data that support the findings of this study are available from the corresponding author upon reasonable request.

## Supplementary Materials

The following are available online at https://www.mdpi.com/article/10.3390/jfmk11020133/s1, Table S1. Standardized 7-Week Motor Learning Program Schedule, Table S2. Task-Specific Criteria for Assessing Phase-Based Motor Learning and Final Performance Quality.
